# Supplementary material for: Employing 11-Ketotestosterone as a Target Analyte for Adrenosterone (11OXO) Administration in Doping Controls
Source: Metabolites. 2024 Feb 26;14(3):141. doi: 10.3390/metabo14030141 (PMC10972305; doi:10.3390/metabo14030141)
Supplement: Supplementary file 1 [file metabolites-14-00141-s001.zip › metabolites-2872061-supplementary.pdf]

Supplementary Materials

Employing 11-Ketotestosterone as Target Analyte for Adrenosterone (11OXO) Administrations in Doping Controls

Thomas Piper, Gregor Fußhöller and Mario Thevis

1) Chemical structures of all employed steroidal compounds

1.1) Target Analytes

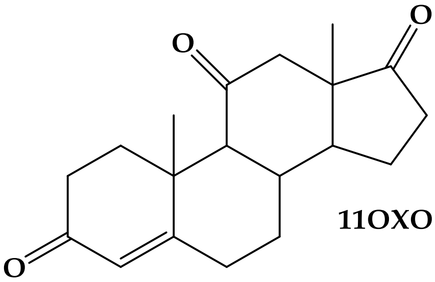

11OXO

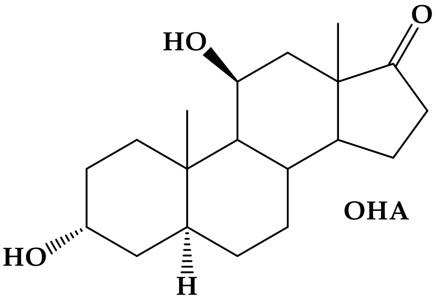

OHA

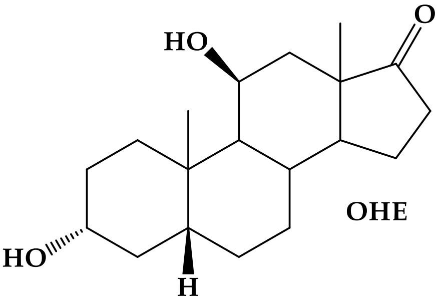

OHE

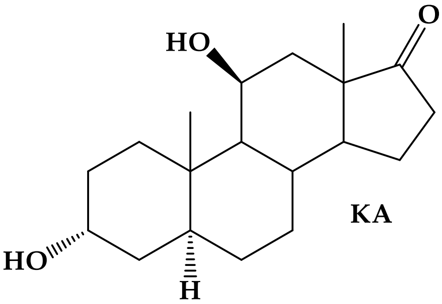

KA

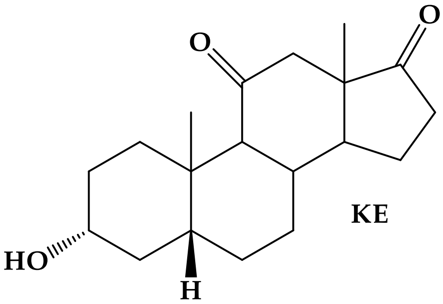

KE

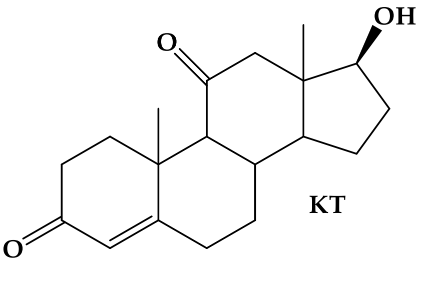

KT

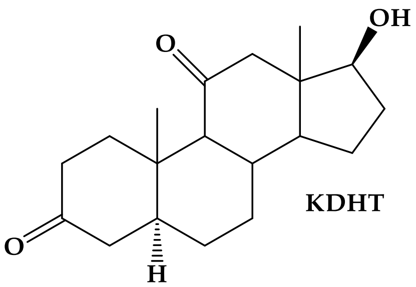

KDHT

1.2) Endogenous reference compound

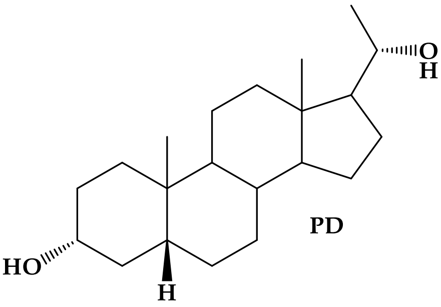

PD

1.3) Internal and reference standards

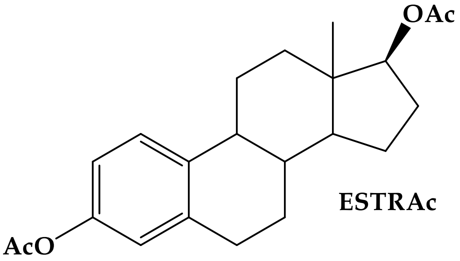

ESTRAc

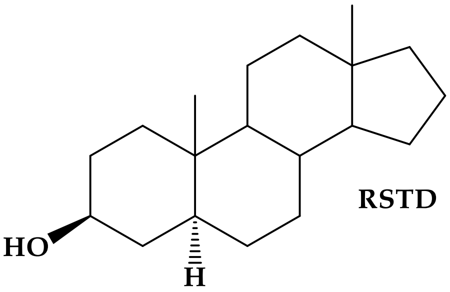

RSTD

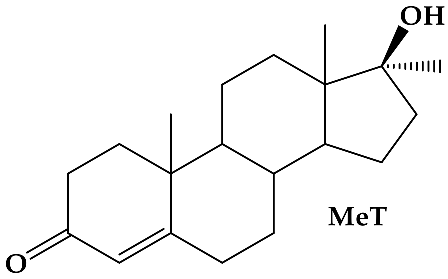

MeT

1.4) Additional steroids under consideration

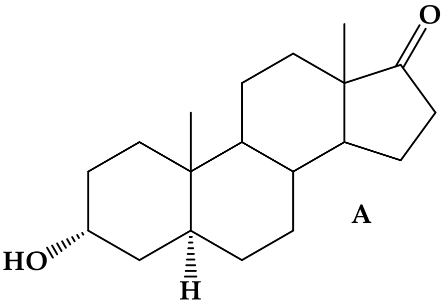

A

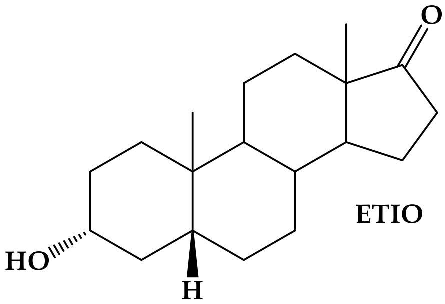

ETIO

Figure S1. Chemical structures of all employed steroidal compounds.
